# European priority review vouchers for neglected disease product development

**DOI:** 10.1136/bmjgh-2023-013686

**Published:** 2024-01-30

**Authors:** David B Ridley, Agustín Martín Lasanta, Ffion Storer Jones, Sarah K Ridley

**Affiliations:** 1 Health Sector Management, Duke University, Durham, North Carolina, USA; 2 Deutsche Stiftung Weltbevölkerung, Brussels, Belgium; 3 Computer Science, Brown University, Providence, Rhode Island, USA

**Keywords:** Health policy, Health economics

## Abstract

**Introduction:**

Neglected diseases are a significant global health challenge. Encouraging the development of therapeutics and vaccines for these diseases would address an important unmet medical need. We propose a priority review voucher programme for the European Union (EU). The developer of a drug or vaccine for a neglected disease would receive a voucher for accelerated assessment of a different product at the European Medicines Agency (EMA).

**Methods:**

This study uses retrospective observational data to estimate the potential commercial value of the proposed voucher programme using a five-step approach: (1) estimating the time saved in the EMA accelerated regulatory review; (2) gauging time reductions in accelerated pricing and reimbursement decisions by EU member states; (3) selecting 10 high-revenue products launched between 2015 and 2020 representing typical voucher users; (4) analysing IQVIA MIDAS sales data for the selected products and (5) calculating the net present value (NPV) of the voucher based on the 10 products.

**Results:**

The accelerated EMA review would reduce regulatory time by an average of 182 days. Additionally, products could save more than a year in many member states through an expedited 120-day pricing and reimbursement review. The estimated NPV of regulatory acceleration by two quarters would be €100 million. In addition, if France, Italy and Spain reviewed pricing and reimbursement in only 120 days, then the value would double.

**Conclusion:**

An EU voucher estimated at more than €100 million, coupled with a US$100 million counterpart, offers a meaningful incentive for novel product development. However, the voucher programme should be part of a comprehensive strategy for tackling neglected diseases, rather than a standalone solution.

**WHAT IS ALREADY KNOWN ON THIS TOPIC**

- Neglected diseases are a significant global health problem that requires the development of drugs and vaccines.

**WHAT THIS STUDY ADDS**

- To encourage product development for neglected diseases, this study proposes a priority review voucher programme for the European Union (EU). Based on the US experience with vouchers, this study estimates the value of an EU voucher.

**HOW THIS STUDY MIGHT AFFECT RESEARCH, PRACTICE OR POLICY**

- This study shows that an EU voucher would substantially increase the reward for product development for otherwise neglected diseases. This study could inspire EU policy-makers just as a previous proposal inspired US policy-makers.

## Introduction

Since 2020, the European Union (EU) has expanded its authority in health matters.^1^ This creates an opportunity to introduce new policy tools. We propose an EU priority review voucher programme to encourage development of new medicines and vaccines (henceforth referred to as ‘products’) for neglected diseases. Neglected diseases are infectious diseases that have historically been neglected by product developers because they are prominent in lower-income countries where people have little ability to pay for new products.^2^

Under the proposed programme, product developers that successfully register a novel therapeutic product addressing a neglected disease (such as tuberculosis, malaria or a tropical disease, as defined by the WHO) would be granted a transferable voucher. This voucher could then be applied for accelerated assessment of another commercially viable product by the European Medicines Agency (EMA).

**Figure 1:**
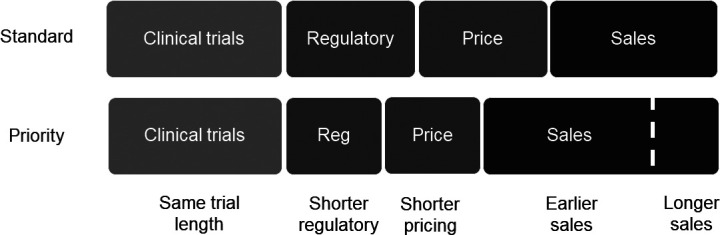
A voucher that shortens regulatory and pricing time creates value through earlier and possibly longer sales.

The proposed programme links two products with each voucher; one product addresses a neglected disease and the other has a high commercial potential. The first product garners a voucher as a reward for countering a neglected disease, while the second product employs the voucher to secure faster regulatory review—referred to as ‘priority review’ in the USA and ‘accelerated assessment’ in the EU.^3^ Expeditious market entry translates into earlier revenue generation (figure 1). By linking the two products, the synergy can render a formerly unprofitable drug economically viable.

The US voucher programme, our basis for this proposal, was created by the US Congress in 2007 to encourage product development for neglected diseases. In 2012, Congress expanded voucher eligibility to rare paediatric diseases, and in 2016 Congress expanded eligibility to medical countermeasures which treat patients in a public health emergency, including a bioterror attack. More than 60 vouchers have been awarded and vouchers sell for about US$100 million each.^4^

The US voucher programme has yielded success. It has helped a non-profit drug developer attract investment for developing a river blindness drug, provided a commercial rationale for continued development of a tuberculosis drug and supported patient access to a Chagas drug through the sale of a voucher.^5^ We anticipate an even stronger incentive with the addition of a similar EU programme.

The programme could also be structured to hasten pricing and reimbursement decisions, although this facet would necessitate engagement and agreement by individual EU member states. Member states are free to set the price of medicines and decide which treatments will be publicly reimbursed. However, member states are expected to make pricing and reimbursement decisions within 180 days of applications according to the Transparency Directive.^6^

Our proposition is a contemporary adaptation of earlier proposals presented for the USA in 2006^2^ and for the EU in 2010.^7^ An update is warranted due to four critical developments. First, the EU’s augmenting involvement in health policies facilitates updated strategies. Second, the US programme’s track record offers valuable insights regarding its efficacy and shortcomings, including product novelty requirements and the need for access-promoting mechanisms. Third, over a decade’s worth of data from the US programme is available, facilitating a more informed estimation of a European voucher’s commercial value. Finally, the recent decrease in US voucher prices, as a consequence of Congressional measures extending eligibility to rare paediatric diseases^8^ and medical countermeasures,^5^ highlights the need for the introduction of an EU voucher to leverage the programme and reinforce incentives.

## Methods

We aimed to assess the potential value of implementing a voucher programme in the EU which is similar and complementary to that of the USA. Our study analysed selected pharmaceutical products that used vouchers in the USA. We estimated the increase in net present value (NPV) from an earlier launch in the EU.

### EMA regulatory review time estimation

We evaluated the potential reduction in time to market by calculating the mean difference between standard and accelerated regulatory review times. We obtained regulatory review time data from the EMA.^9^ We included only products receiving positive opinions. The data included active time, clock stops and authorisation time. Active time is the period in which the scientific evaluation of a medicine is carried out. Clock stop is a period during the drug evaluation process where the regulator pauses the review timeline while the developer assembles more information. Under accelerated assessment, a maximum of 1 month of clock stop can be granted to the applicant. If it requires longer, the initial marketing authorisation reverts to a standard timetable. Under the standard timetable, the applicant can be granted a 3-month clock stop, extendable up to 6 months in total. In exceptional cases, longer clock stops may be granted, such as when there is a need for inspections or scientific advice. Authorisation time is the period in which the regulator completes the review and decides whether to authorise the product.

### Pricing and reimbursement time estimation

We analysed the potential time savings from expedited pricing and reimbursement decisions within EU member states, using data from the European Federation of Pharmaceutical Industries and Associations survey (2017–2020).^10 11^ The data included the time between marketing authorisation and product availability to patients in all EU member states except Cyprus, Luxembourg and Malta. We omitted those three member states from the analysis. We estimated potential time savings if reimbursement delays were reduced to 120 days for brand name products, as proposed by the European Commission.^12^ We considered time savings from all member states and time savings from just three member states. We converted decision times to quarters for consistency with the sales data.

### Product selection criteria

We selected pharmaceutical products that used a voucher in the USA between the first quarter of 2007 and the first quarter of 2020, were approved by the EMA by the first quarter of 2020, had positive sales in our data in at least five EU member states in 2021 and used a voucher for their first indication. Of the 22 products that we found that met the first criterion, 10 satisfied all the criteria and were included in the analysis (online supplemental appendix table A1).

10.1136/bmjgh-2023-013686.supp1 Supplementary data

### Sales data analysis

We used IQVIA MIDAS sales data to analyse sales by compound, country and quarter from the first quarter of 2015 to the first quarter of 2022. We included 21 EU countries: Austria, Belgium, Croatia, Czechia, Estonia, Finland, France, Germany, Greece, Hungary, Ireland, Italy, Latvia, Lithuania, Netherlands, Poland, Romania, Slovakia, Slovenia, Spain and Sweden. Due to unavailability of sales data, we excluded Bulgaria, Denmark and Portugal. Cyprus, Luxembourg and Malta also lacked sales data but had already been excluded from the analysis due to missing pricing and reimbursement data.

For product-country pairs with at least two quarters of sales data, we projected missing quarters through quarter 24 using industry average growth rates^13^ (online supplemental appendix table A2). For sales from quarters 25 to 52, we assumed flat sales equal to quarter 24. For quarters beyond 52, we assumed zero sales without a voucher,^14^ but extra quarters of sales with a voucher according to the number of quarters of acceleration. If a product-country pair had less than two quarters of sales data, we omitted it.

### NPV calculation

We assumed that the voucher programme would expedite sales and provide a competitive benefit against other brands due to earlier launch relative to them.^15^ We also considered cases in which the voucher created additional quarters with sales by allowing earlier launch and the same generic entry.

IQVIA reported sales in US$ which we converted into € using an exchange rate of $1 to €0.9, which was the average exchange rate from 2019 to 2021. We calculated net sales (*N*) after rebates, cost of goods and taxes. We assumed rebates of 25%, because rebates for European countries are typically 20%–29%.^16^ We assumed a cost of goods of 20%, because generic drugs are often about 20% of brand prices in competitive markets in which price would be close to cost. We assumed a marginal corporate tax rate of 21%, because that is the US tax rate and the USA is home to many drug makers. A company could pay a lower rate if its assets were in a country with a lower rate, such as Ireland at 13%, or if it were a young company with high development costs relative to revenue.

We assumed that all products using vouchers are approved, because drug makers use vouchers for products with the highest expected value. Indeed, under the current US voucher system in place since 2014, we know only one voucher-using product which was not approved by the Food and Drug Administration (FDA). However, the product was approved by the EMA.

We calculated the NPV (*V*) of sales for a product. We assumed an annual opportunity cost of capital of 10.5%^17^ which implies a quarterly cost of capital (*i*) of 2.5% (online supplemental appendix table A3). We assumed six quarters between voucher award and submission ($\tau_{s}$).^18^ We assumed a competitive benefit (*B*) of 3.6% per quarter. We rounded the extra time (∆τ_e_+∆τ_p_) to the nearest integer. The NPV of sales depends on regulatory time at the EMA ($\tau_{e}$), as well as pricing and reimbursement time ($\tau_{p}$):

$$V=\sum_{t=1}^{52+∆\tau_{e}+∆\tau_{p}}\frac{(1+B)N_{t}}{{\left(1+i\right)}^{\left(t+\tau_{s}+\tau_{e}+\tau_{p}\right)}}$$

### Voucher value calculation

The value of the voucher was the difference between NPV calculations for standard and accelerated times. We estimated NPV for each drug in each country. We then aggregated. The online supplemental file 1 contains more detail about the assumptions and methods.

### Patient and public involvement

Patients and the public were not involved in this research study.

## Results

### EMA regulatory review time

The mean standard review time was 412 days from 2017 to 2020 (table 1). The mean accelerated review time was 230 days. The difference was 182 days. If we exclude clock stops, then the mean difference was 66 days.

**Table 1:** Regulatory review time by the EMA for standard and accelerated products for 2015–2021

	Standard (days)				Accelerated (days)				
**Year**	**Active**	**Clock stop**	**Authorisation**	**Sum**	**Active**	**Clock stop**	**Authorisation**	**Sum**	**Saved**
2015	202	134	61	396	151	37	57	245	151
2016	200	146	62	408	150	35	55	240	168
2017	188	172	62	422	136	37	43	215	207
2018	195	180	59	434	143	51	33	227	208
2019	193	168	59	420	148	47	31	226	194
2020	192	160	60	412	144	42	39	225	187
2021	185	154	54	393	143	49	42	234	159
Mean	194	159	60	412	145	43	43	230	182

Source: authors’ calculations using EMA data from 2015 to 2021.

EMA, European Medicines Agency.

### Pricing and reimbursement time

We report average times for pricing and reimbursement among member states between 2017 and 2020 (online supplemental appendix table A4). In Germany, the median was 53 days and the mean was 133 days. In France, Italy and Spain, the other largest pharmaceutical markets in the EU, the median and mean times exceeded 1 year.

### Sales

**Figure 2:**
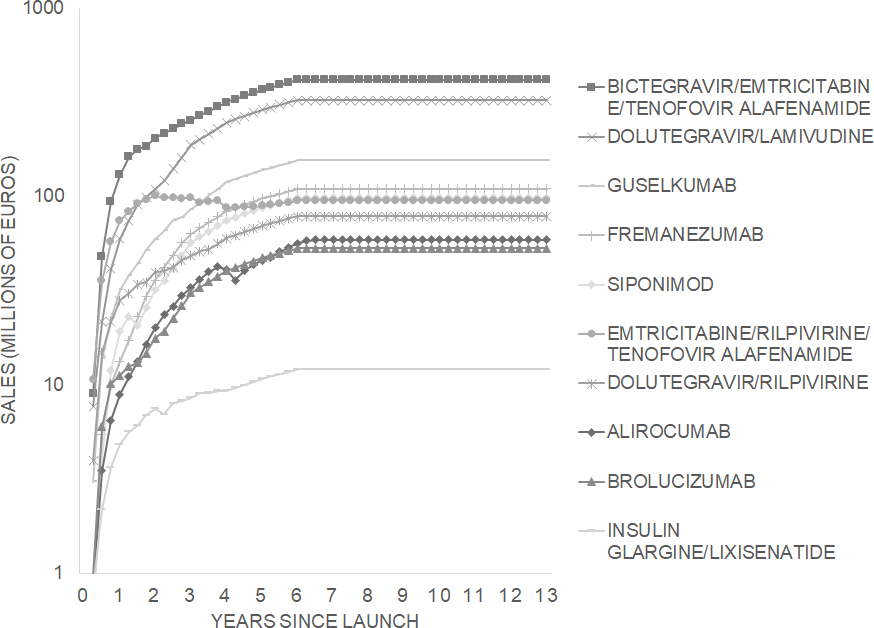
Projected quarterly sales in 21 EU member states since launch for 10 products that used vouchers in the USA. Note: the vertical axis uses a log scale, so each tick is greater than the previous tick by a factor of 10.

We used IQVIA sales data for 10 products in 21 EU member states from 2015 to the first quarter of 2022. Among the 10 products, projected EU peak quarterly sales ranged from about €10 million for a diabetes drug (insulin glargine/lixisenatide) to more than €400 million for an HIV drug (bictegravir/emtricitabine/tenofovir alafenamide) (figure 2).

### Voucher value

We estimate that the voucher would have had a median value of about €100 million for the 10 products we study, assuming a two-quarter acceleration (table 2). France, Germany, Italy and Spain account for most of the value of a voucher (online supplemental appendix table A5). The voucher value would double to about €200 million for the 10 products we study if a voucher had accelerated not only regulatory review but also accelerated a pricing and reimbursement decision to 120 days in France, Italy and Spain. We exclude Germany here, because its pricing and reimbursement decisions are already fast.

**Table 2:** Value of a voucher by product under various scenarios for regulatory and reimbursement times

			Voucher net present value (millions of Euros)			
**Molecule name**	**Brand name**	**Therapeutic class**	**Same R; early G**	**Same R; same G**	**120-day R top 3; same G**	**120-day R all; same G**
Alirocumab	Praluent	Lipid-modifying drug	44	54	101	143
Bictegravir/emtricitabine/tenofovir alafenamide	Biktarvy	Antiviral	335	411	1022	1107
Brolucizumab-dbll	Beovu	Antineovascularisation drug	42	52	72	117
Dolutegravir/lamivudine	Dovato	Antiviral	248	307	769	838
Dolutegravir/rilpivirine	Juluca	Antiviral	64	78	213	220
Emtricitabine/rilpivirine/ tenofovir alafenamide	Odefsey	Antiviral	97	115	275	294
Fremanezumab-vfrm	Ajovy	Antimigraine drug	83	103	178	272
Guselkumab	Tremfya	Immunosuppressant	122	151	274	346
Insulin glargine/lixisenatide	Soliqua	Insulin	10	13	16	33
Siponimod	Mayzent	Immunosuppressant	76	94	179	222
Median			79	98	196	247

The net present value of a voucher depends on whether generic drug entry (‘G’) is early or the same, and whether reimbursement decisions for France, Italy and Spain (‘R’) are early or the same. Times include time saved from reduced clock stops. Source: Authors’ calculations using data from IQVIA, European Medicines Agency and European Federation of Pharmaceutical Industries and Associations.

## Discussion

The value of a priority review voucher depends on how much time can be saved. We found that the mean standard review time was four quarters and the mean accelerated review time was two. To estimate the value of a voucher in Europe, we selected 10 products that had used vouchers in the USA and were approved by the EMA. We used IQVIA MIDAS sales data and adjusted sales for rebates and taxes. We estimated a median voucher value of about €100 million for the 10 products analysed, assuming a two-quarter acceleration. This value could double if the voucher also expedited pricing and reimbursement decisions in key markets.

### Limitations

Our analysis has limitations in accurately estimating the median value of a voucher. The estimated value may be underestimated if (1) developers optimise product selection for the EU market, (2) inclusion of the five missing countries significantly increases sales, (3) products maintain positive sales beyond the 12th year, (4) companies leverage lower tax jurisdictions, (5) rebates are smaller than 25% or (6) there is a low supply of vouchers (which moves the voucher price up the demand curve). Conversely, the value may be overestimated if (1) a high number of vouchers are awarded, diluting their worth, (2) rebates are larger than 25%, (3) accelerated approval does not reduce clock stop time or (4) the EMA streamlines its standard regulatory review process, reducing the relative advantage of vouchers.

The estimates in this analysis are imprecise. Nevertheless, the analysis illustrates what factors to consider when calculating a voucher’s value. Furthermore, the analysis indicates that the voucher could have enough value to have policy significance and real-world impact in conjunction with other mechanisms.

An EU voucher scheme would also have limitations like its US counterpart.^2 5 19 20^ First, the voucher’s value could be too generous, rewarding research that would have been conducted without the stimulus.^21^ Hence, policy-makers should adopt stringent eligibility criteria as outlined in our discussion on voucher-receiving products. Second, the voucher scheme might be insufficient. A voucher worth €100 million is an order of magnitude less than the full cost of drug development from start to finish.^17^ Hence, the voucher could pull through drugs that had already started development. Also, the voucher system should not be a substitute for other incentives, but rather part of a comprehensive strategy to stimulate development.^5^ Third, vouchers do not guarantee access to therapies.^5 19 20^ We recommend a requirement for developers to provide detailed access plans, as delineated in our discussion section. Fourth, an accelerated review process could strain the resources of the EMA.^2^ To address the added burden, US voucher holders are required to pay a user fee, which could be a viable strategy in the European context as well. Finally, expedited assessment might compromise safety, given that regulators would have less time to scrutinise the required documentation.^22^ However, there has been no evidence of quality compromise within the US voucher programme.

### Voucher-receiving products

We recommend that eligibility for voucher-receiving products be restricted to novel drugs having clinical superiority and significant therapeutic benefits compared with existing treatments. The primary active ingredient should not have been approved in any product prequalified by the WHO or authorised by a WHO listed authority, such as the FDA,^23^ for more than 2 years prior to the EU voucher application submission. For combination therapies, at least one active ingredient should meet this criterion.

Developers should be required to submit to the EMA a global access plan, including pricing, manufacturing sites and target markets at the time of product submission. If a voucher is granted, the EMA should publicly disclose the access plan to ensure developer accountability. This is analogous to the access mechanism in place for the vector expedited review voucher in the USA.^24 25^

**Figure 3:**
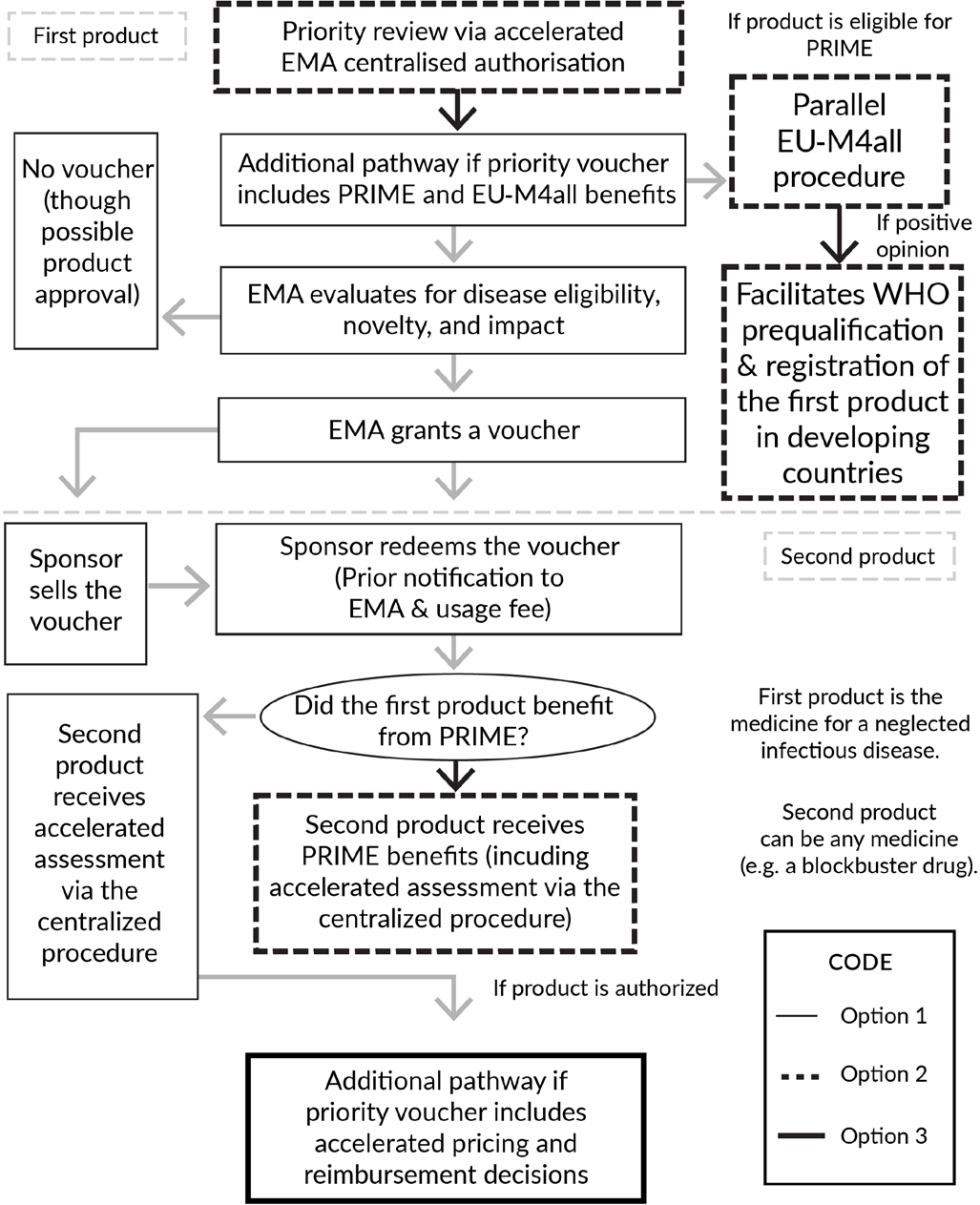
Decision tree of the EU priority review voucher. EMA, European Medicines Agency; EU-M4all, EU-Medicines for All; PRIME, PRIority MEdicines.

Moreover, the EU could grant voucher recipients additional benefits such as the PRIority MEdicines (PRIME) scheme for enhanced interaction with the EMA and EU-Medicines for All (EU-M4all), which fosters collaboration between the EMA, WHO and regulatory authorities from third countries for product approval beyond the EU. Figure 3 illustrates how these options interact. Option 1 shows the regulatory pathway of the voucher programme in the EU, option 2 if it had PRIME and EU-M4all benefits and option 3 if it granted accelerated pricing and reimbursement.

We recommend that the EMA only award vouchers to products for neglected diseases. This would be a narrower scope than current US law, which includes not only products for neglected diseases, but also rare paediatric diseases and medical countermeasures. We recommend focusing on neglected diseases because of their large global disease burden and because products for these diseases have no potential for high prices. Furthermore, the voucher would complement EU instruments that fund preclinical and clinical research in neglected diseases such as the European & Developing Countries Clinical Trials Partnership (EDCTP). Finally, we recommend narrow eligibility to reduce the voucher supply, which raises the voucher price and increases the incentive.^26^ An alternative to narrow eligibility would be an arbitrary cap on the number of vouchers. However, a cap introduces uncertainty, which negates the benefit of the cap.

We expect that two EMA vouchers could be awarded annually, assuming that the EU voucher programme may stimulate further investment in neglected diseases. This is double the US average of one voucher per year between 2015 and 2022.

### Voucher-using products

Entities intending to use a voucher should notify the EMA at least 90 days in advance. They should also pay an additional user fee, akin to the US$1.5 million fee in the USA in 2023.^27^ This can help offset the programme’s costs. Limiting voucher eligibility will also reduce strain on the EMA.

It is essential to distinguish priority review vouchers from exclusivity vouchers, which delay the introduction of generic alternatives.^28–31^ Priority review vouchers expedite the review process but do not extend market exclusivity. In fact, in some instances, the shortened regulatory timeline could result in reduced exclusivity.

## Conclusions

We advocate for the implementation of a priority review voucher programme in the EU to stimulate the development of novel products for neglected diseases. The voucher programme would provide an additional benefit to patients in Europe: earlier access to products that use vouchers.

The incentive offered by an EU voucher would be comparable to a US voucher. While the US market is somewhat more lucrative per quarter of sales, the EU voucher would provide more quarters saved.

An EU voucher programme would provide a substantial incentive. The combined value of the US and EU vouchers would be about €200 million which investors say would be a meaningful incentive.^5^ If member states also accelerated pricing and reimbursement decisions for voucher-using products, then the value would be higher. Nevertheless, the burden of neglected diseases is massive and warrants additional incentives, including more research funding,^32^ development funding,^33 34^ monetary prizes^35^ and advance market commitments.^36^

## Data Availability

Data may be obtained from a third party and are not publicly available. MIDAS sales data can be obtained through the purchase of a licence from IQVIA. Data pertaining to the timing for regulatory review, pricing and reimbursement can be downloaded from EMA and European Federation of Pharmaceutical Industries and Associations as referenced in the article. Alternatively, the timing data can be requested from the corresponding author.
