# Supplementary material for: European priority review vouchers for neglected disease product development
Source: BMJ Glob Health. 2024 Jan 30;9(1):e013686. doi: 10.1136/bmjgh-2023-013686 (PMC10828857; doi:10.1136/bmjgh-2023-013686)
Supplement: Supplementary data [file bmjgh-2023-013686supp001.pdf]

SUPPLEMENTARY INFORMATION

Table A1: Products excluded from the analysis despite using vouchers in the US

Molecule Name	Brand Name	Therapeutic class	Excluded because late EU launch, if at all	Excluded because voucher used for second or later indication
Calcium/magnesium/potassium/ sodium oxybates	Xywav	Nervous system drug	Yes	
Canakinumab	Ilaris	Immuno-suppressant		Yes
Emtricitabine/tenofovir alafenamide	Descovy	Antiviral		Yes
Filgotinib	Jyseleca	Immuno-suppressant	Yes	
Ravulizumab-cwvz	Ultomiris	Immuno-suppressant	Yes	
Rimegepant	Nurtec ODT	Anti-migraine drug	Yes	
Ruxolitinib	Jakafi	Antineoplastic drug		Yes
Semaglutide	Rybelsus	Blood glucose lowering drug		
Semaglutide	Wegovy	Antiobesity drug	Yes	Yes
Tirzepatide	Mounjaro	Blood glucose lowering drug	Yes	
Treprostinil	Tyvaso DPI	Antithrombotic drug	Yes	Yes
Upadacitinib	Rinvoq	Immuno-suppressant		Yes

Source: Authors’ analysis using data from the European Medicines Agency and the Food and Drug Administration.

Table A2: Quarterly growth rate

Year	Share of peak (1)	Implied annual growth rate	Implied quarterly growth rate
1	11%		
2	31%	182%	30%
3	58%	87%	17%
4	76%	31%	7%
5	89%	17%	4%
6	100%	12%	3%

Source: Authors’ estimates based on Robey and David (2016).(1)

Ridley DB, *et al. BMJ Glob Health* 2024; 9:e013686. doi: 10.1136/bmjgh-2023-013686

Table A3: Variables

Notation	Variable	Value	Source
B	Competitive effect	Varies by time saved	See equation 2
c	Cost of goods sold	0.2	Assumption
i	Depreciation rate (quarterly)	0.025	10.5% annual rate (2)
x	Exchange rate (€/USD)	0.9	Average for 2019-2021
g	Growth rate	See Table A2	Robey and David (2016)(1)
m	Marginal tax rate	0.21	US corporate income tax rate
V	Net present value (Euros)	Varies by drug	See equation 3
N_t	Net sales (Euros)	Varies by country, drug, and quarter	See equation 1
$\Delta\sigma$	Peak share per quarter early	0.009	Regnier and Ridley (2015)(3)
σ	Peak share without early entry	0.25	Assumption
s_t	Sales (USD)	Varies by country, drug, and quarter	IQVIA MIDAS
t	Quarter	Varies by quarter	
r	Rebate	.25	Drug rebates for European countries are typically 20-29% (4)
τ_s	Time before submission (quarters)	6	Median for 16 drugs in 2020 GAO report (5)
τ_e	Time for EMA (quarters)	See Table 1	EMA data for 2015-2021
τ_p	Time for pricing (quarters)	See Table A4	EFPIA data for 2017-2020
$\Delta\tau_e$	Change in time for EMA (quarters)	Varies by type	Assumption
$\Delta\tau_p$	Change in time for pricing (quarters)	Varies by country	Assumption

Source: Authors

Voucher value calculation details

We calculated net sales in a quarter (N_t) using sales (s_t) and accounting for the cost of goods sold (c), the rebate (r), the marginal tax rate (m), and the exchange rate (x).

$$N_t = (1 - c - r)\,(1 - m)\,x\,s_t \tag{1}$$

We then calculated the competitive benefit (B) due to an early mover advantage of taking sales from competitors. We added the time saved in regulatory time ($\Delta\tau_e$) and pricing and reimbursement time ($\Delta\tau_p$). We then multiplied the time saved by the increase in sales per quarter early ($\Delta\sigma/\sigma$).

$$B = (\Delta\tau_e + \Delta\tau_p)\Delta\sigma/\sigma \tag{2}$$

We then calculated the net present value of sales (V). We summed net sales (N_t) across quarters (t) while accounting for the competitive benefit (B). We accounted for the time value of money which depends on the interest rate (i), as well as the time to submission (τ_s) as well as the regulatory (τ_e) and reimbursement times (τ_p).

$$V = \sum_{t=1}^{52+\Delta\tau_e+\Delta\tau_p} \frac{(1+B)N_t}{(1+i)^{(t+\tau_s+\tau_e+\tau_p)}} \tag{3}$$

More details about the variable definitions and data sources appear in Appendix Table A3.

Ridley DB, *et al. BMJ Glob Health* 2024; 9:e013686. doi: 10.1136/bmjgh-2023-013686

Table A4: Pricing and reimbursement time by EU member states for 2017 to 2020

Country	Median (days)	Mean (days)	Time savings from median if 120 days
Austria	266	315	146
Belgium	417	534	297
Bulgaria	719	764	599
Croatia	419	479	299
Czech Republic	532	573	412
Denmark	101	176	0
Estonia	518	599	398
Finland	333	396	213
France	442	497	322
Germany	53	133	0
Greece	453	498	333
Hungary	383	480	263
Ireland	448	541	328
Italy	372	429	252
Latvia	611	627	491
Lithuania	536	594	416
Netherlands	231	294	111
Poland	758	844	638
Portugal	625	676	505
Romania	875	899	755
Slovakia	522	564	402
Slovenia	505	577	385
Spain	468	517	348
Sweden	183	261	63

Source: Authors' calculations using data from the EFPIA Patients W.A.I.T. Indicator Survey from 2017 to 2020.(6,7)

Table A5: Median value of a voucher by country under various scenarios for regulatory and reimbursement times

	Voucher net present value (millions of Euros)		
**Country**	**Early G; same R**	**Same G; same R**	**Same G; 120-day R all**
Austria	2.1	2.6	5.0
Belgium	3.7	4.4	12.5
Croatia	0.8	0.9	2.7
Czechia	1.3	1.6	6.1
Estonia	0.1	0.1	0.4
Finland	0.3	0.4	0.8
France	16.9	20.8	63.8
Germany	15.8	18.8	18.8
Greece	0.1	0.2	0.5
Hungary	0.3	0.4	1.0
Ireland	1.5	1.8	5.6
Italy	17.5	21.6	55.8
Latvia	0.1	0.2	0.7
Lithuania	0.1	0.2	0.6
Netherlands	3.5	4.3	7.1
Poland	0.5	0.6	3.3
Romania	1.6	1.9	11.8
Slovakia	0.6	0.8	2.7
Slovenia	0.4	0.5	1.7
Spain	19.8	23.9	76.7
Sweden	1.1	1.4	1.9
Total	88.1	107.4	279.7

Notes: The net present value of a voucher depends on whether generic drug entry (“G”) is early or the same, and whether reimbursement decisions (“R”) are early or the same. Times include time saved from reduced clock stops. Source: Authors’ calculations using data from IQVIA, EMA, and EFPIA.

References

1. Robey S, David FS. Drug launch curves in the modern era. Nat Rev Drug Discov. 2016;16(1):13–4.

2. DiMasi JA, Grabowski HG, Hansen RW. Innovation in the pharmaceutical industry: New estimates of R&D costs. J Health Econ. 2016;47:20–33.

3. Regnier SA, Ridley DB. Market watch: Forecasting market share in the US pharmaceutical market. Vol. 14, Nature Reviews Drug Discovery. 2015. p. 594–5.

4. Vogler S, Zimmermann N, Babar ZUD. Price comparison of high-cost originator medicines in European countries. Expert Rev Pharmacoeconomics Outcomes Res. 2017;17(2).

5. Government Accountability Office. FDA's Priority Review Voucher Programs [Internet]. Washington, DC; 2020 [cited 2020 May 15]. Available from: https://www.gao.gov/assets/710/704207.pdf

6. Newton M, Scott K, Troein P. EFPIA Patients W.A.I.T. Indicator 2021 Survey. IQVIA [Internet]. 2022;(April). Available from: https://www.lif.se/globalassets/pdf/rapporter-externa/wait/efpia-patients-w.a.i.t.-indicator-2022.pdf

7. Newton M, Scott K, Troein P. EFPIA Patients W.A.I.T. Indicator 2021 Survey. IQVIA [Internet]. 2022;(July). Available from: https://www.efpia.eu/media/676539/efpia-patient-wait-indicator_update-july-2022_final.pdf
